# Smoking index, lifestyle factors, and genomic instability assessed by single-cell gel electrophoresis: a cross-sectional study in subjects from Yucatan, Mexico

**DOI:** 10.1186/s13148-019-0745-7

**Published:** 2019-10-29

**Authors:** Alejandra Locken-Castilla, Elda Leonor Pacheco-Pantoja, Fátima Rodríguez-Brito, Sherlin May-Kim, Victor López-Rivas, Angel Ceballos-Cruz

**Affiliations:** 1 Medicine School, Health Sciences Division, Universidad Anáhuac Mayab, Km 15.5 Carr. Mérida-Progreso, Mérida, Yucatán México; 2 Health Sciences Division, Universidad Anáhuac Mayab, Km 15.5 Carr. Mérida-Progreso, Mérida, Yucatán México

## Abstract

**Background:**

It is widely accepted that genomic instability is associated with several mechanisms involving oxidative stress, which can increase the rate of DNA breaks. Such factors include smoking, impairments in body composition, an unhealthy lifestyle, and a hereditary history of cancer. The aim was to evaluate the degree of association of genomic instability in smokers and non-smokers, and how the risk could change depending on the lifestyle and other causes. For this purpose, a survey of tobacco consumption, dietary patterns, physical activity, antecedents of cancer, and body composition assessment was carried out. Genomic instability was evaluated through a single-cell gel electrophoresis using peripheral blood mononuclear cells in three different conditions of oxidative stress. The analysis of genomic damage degree was performed through a dimension reduction procedure (principal component analysis) from 16 parameters per treatment (adding up 48 parameters of genomic damage per subject) and a binary logistic regression model for DNA fragmentation risk.

**Results:**

The sample consisted of 82 participants, divided into three age groups: young adults (18–35 years), adults (36–59 years), and older adults (60–95 years). As expected, the results showed a significant positive correlation of age with genomic damage rates, represented by 2 PCA groups (*p* = 0.027, *p* = 0.004). There were consistent significant positive associations of genomic damage rates with smoking index and three PCA groups (*p* = 0.007, *p* = 0.004, *p* = 0.009). The smoking status and age group analysis revealed that there were significant differences for adult smokers with the same aforementioned PCA groups (*p* = 0.002, *p* = 0.001, *p* = 0.010). In addition, higher DNA damage rates were found in subjects with incorrect diet patterns, long sitting hours, and previous exposure to radiation. The analysis with binary logistic regression displayed two models in which lifestyles (age, diet, and/or sedentarism) did not change the significance of smoking index for DNA fragmentation risk; however, when physical activity was present in the model, the smoking index was not a significant factor for DNA damage risk.

**Conclusions:**

Although it is well known that smoking affects human health in different ways, DNA fragmentation can be analyzed by a damage phenotypic analysis and integrate a risk analysis reshaped by diet and lifestyle in general.

## Background

The global tobacco epidemic is responsible for more than seven million deaths each year. The World Health Organization (WHO) has proposed specific measures for every country to protect their population from this threat, which is one of the biggest single preventable causes of death [1]. Nowadays, it is widely accepted that smoking produces obstructive lung disease, predisposes to respiratory tract infections, and increases the risk of neoplasms in the airway and other peripheral tissues (tongue, colon, cervix, etc.) [2]*.* In spite of this, and public institutions’ efforts to ban the consumption of cigarettes in public spaces, the rates of smoking have not decreased and, particularly, in Mexico, the smoking rate reaches 17.6% of the population. Even more alarming is that 98.4% of smokers are conscious about the harmful (and potentially fatal) consequences of this habit [3]. It is noteworthy to mention that smoking is one of the six modifiable factors in the “25 × 25 program” which, by 2025, aims to reduce non-communicable disease mortality by 25% from the levels reached in 2010. This includes cardiovascular disease, chronic respiratory diseases, cancers, and diabetes [4].

The association of smoking and genomic damage has been explored previously, identifying a connection with some specific compounds present in different type of cigarettes [5], as well as different types of tobacco [6]. However, the clinical importance of this connection (smoking and DNA damage) is still a topic of ongoing research. On the other hand, it has been proposed that epigenetic alterations could explain many of the manifestations of the deleterious effects of smoking, even at a metabolic level, with changes, for example, in the adipose tissue [7]. These epigenetic changes may arise from inheritance and lifestyle factors (nutrition, physical activity, addictions). The single cell gel electrophoresis (SCGE) or comet assay is a technique that provides a rapid analysis and is recognized as a sensitive bio-indicator of genomic damage [8]. This assay has proven to be useful throughout the years in the study of genotoxic effects of pollutants, pesticides, and other substances either in animal or plant cells/tissues [9]. SCGE has been used in clinical setting to investigate the existence of concurrent genomic damage in different illnesses (asthma, cervical dysplasia, diabetes mellitus type 2, malnutrition, infectious diseases, among others) [10] and to screen the extent of damage caused by chemotherapeutic agents [11]. As for studying the habit of smoking, SCGE has been used to demonstrate genomic instability (DNA damage) attributable to cigarette smoking, but there have been inconsistencies in the reported results, as they generally vary depending on the score methodology used to grade genome fragmentation [12].

In Mexico, there are very few studies specifically dedicated to analyzing the genomic damage of smoking; we only found four of them that have taken a history of smoking into account. Two of those [13, 14] were related to the use of pesticides and its association with genotoxicity, where smoking was included as a possible confounding variable. None of them found a significant association either with micronuclei assay (another procedure for phenotypic study of genomic instability) or with SCGE. The other two studies explored the direct connection between smoking and genomic damage in buccal cells using SCGE, with a small sample size (*n* = 20) [15] and micronuclei frequency in lymphocytes [16], with significant and non-significant results for association to smoking, respectively.

In the present investigation, we aimed to evaluate genomic damage in peripheral blood mononuclear cells (PBMCs), in terms of basal DNA damage and oxidative stress-induced damage (H_2_O_2_ treatment), using image analysis for SCGE, in current and former smokers, as well as non-smokers. The analysis also included those factors that could exert some epigenetic regulation, like diet, exercise, nutritional status, lifestyle habits, and a family history of cancer. In order to make the analysis of genomic damage more inclusive, we integrated a principal component analysis procedure (PCA) and a model of binary logistic regression with the aforementioned variables. We hypothesized that genomic instability caused by smoking is epigenetically regulated by other factors that modify the extent of the damage.

## Results

### Study design

Data in this study were drawn from a sample of 82 volunteer participants, aged 18–95 years old. The sample was divided into three age categories and defined as follows: young adults (18 to 35 years), adults (36 to 59 years), and older adults (60 years and older).

The groups were composed of 26, 32, and 24 participants, respectively. All of them were interviewed and requested to sign the informed consent form.

Once the form was completed, the subjects were asked to answer a specially designed questionnaire, composed of five domains: smoking history, diet, physical activity, family history of cancer, and miscellaneous data. Also, the nutritional status and body composition were recorded. To analyze genomic damage, a venous blood sample was collected and processed immediately. Our sample was composed of about half being smokers (*n* = 39) and half being non-smokers (*n* = 43). Furthermore, the smoker group was split into current smokers and former smokers (Table 1).

**Table 1:** Characteristics of the study population

	Smokers		Non-smokers	Total
	Current smokers	Former smokers		
Participants (%)	22 (26.8%)	17 (20.7%)	43 (52.4%)	82 (100%)
Men (%)	12 (14.6%)	11 (13.4%)	12 (14.6%)	35 (42.7%)
Women (%)	10 (12.2%)	6 (7.3%)	31 (37.8%)	47 (57.3%)
Age mean	40.6	55.3	47.2	47.1
(95% CI)	(31–50.2)	(45–65.6)	(41–53.4)	(42.5–51.7)
Range	18–89	19–95	18–93	18–95
Age				
Young adults group (18–35 years)	11 (13.4%)	3 (3.7%)	12 (14.6%)	26 (32%)
Adults group 2 (36–59 years)	6 (7.3%)	7 (8.5%)	19 (23.2%)	32 (39%)
Older adults group 3 (60–95 years)	5 (6%)	7 (8.5%)	12 (14.6%)	24 (29%)
BMI				
Normal	6 (7.3%)	6 (7.3%)	9 (11%)	21 (25.6%)
Overweight	10 (12.2%)	7 (8.5%)	19 (23.2%)	36 (43.9%)
Obesity	6 (7.3%)	4 (4.9%)	15 (18.3%)	25 (30.5%)
Passive smokers	N/A	7 (8.5%)	8 (9.7%)	15 (18.2%)
Non-passive	N/A	10 (12.2%)	35 (42.6%)	45 (54.8%)
Diet				
Correct	9 (11%)	10 (12.2%)	30 (36.6%)	49 (59.8%)
Incorrect	13 (15.8%)	7 (8.5%)	13 (15.8%)	33 (40.2%)
Regular exercise	14 (17%)	11 (13.4%)	35 (42.6%)	60 (73%)
Family history of cancer (grandparents)	4 (4.9%)	5 (6%)	16 (19.5%)	25 (30.4%)
Family history of cancer (parents)	5 (6%)	4 (4.9%)	10 (12.2%)	19 (23.1%)

Data are presented as the number and percentage of participants; age is the mean and corresponding confidence intervals. 95% CI = 95% confidence interval

The OpenComet software retrieved 16 parameters per treatment (control, 5% and 10% of H_2_O_2_) to add up 48 variables of genomic damage per case.

A dimension reduction was carried out with a PCA procedure, obtaining scores (coefficients) that were used to perform all the comparisons and correlations. The PCA procedure subsequently loaded into the following genomic damage indexes: comet, head, and tail, with corresponding treatment groups: control, 5% (T5) and 10% (T10) of H_2_O_2_. The analysis is described for age, each surveyed domain, and body composition.

### Genotoxicity and age

**Fig. 1:**
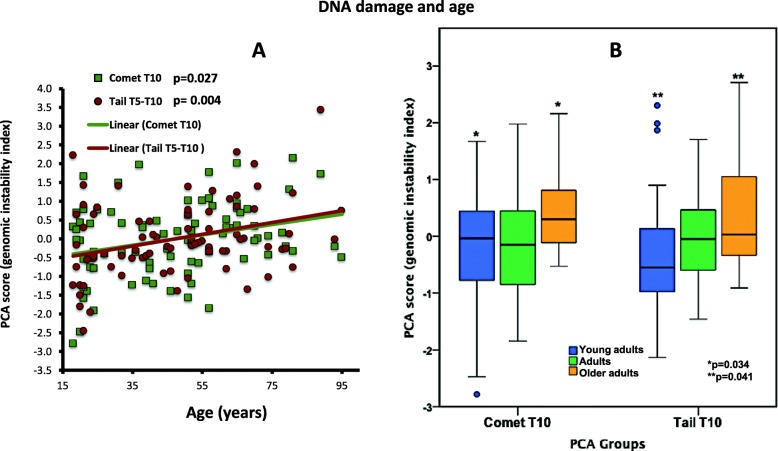
DNA damage and age. **a** PCA group involving body comet (green squares) (*r* = .246, *p* = 0.027), and PCA group related to Tail length (red circles) (*r* = .313, *p* = 0.004), displayed positive significant correlation with age. **b** DNA damage was higher in older adults. Two PCA groups (comet, *p* = 0.034 and tail, *p* = 0.041), exhibited higher significant rates of DNA fragmentation in older adults (orange bars) group when compared with young adults (blue bars). Adults (age middle group, green bars) did not show any significant differences with the rest of the groups

Two PCA groups showed positive statistically significant correlations for age and DNA damage: comet T10 (*r* = .246, *p* = 0.027) and tail T5-T10 (*r* = .313, *p* = 0.004) (Fig. 1a).

One-way ANOVA with subsequent post hoc *t* tests among the different groups confirmed differences within the age group categories. Two PCA groups (comet T10 and tail T10) showed a higher significant difference (*p* = 0.034, *p* = 0.041, respectively) between older adults versus young adults (Fig. 1b).

### Genotoxicity and smoking

**Fig. 2:**
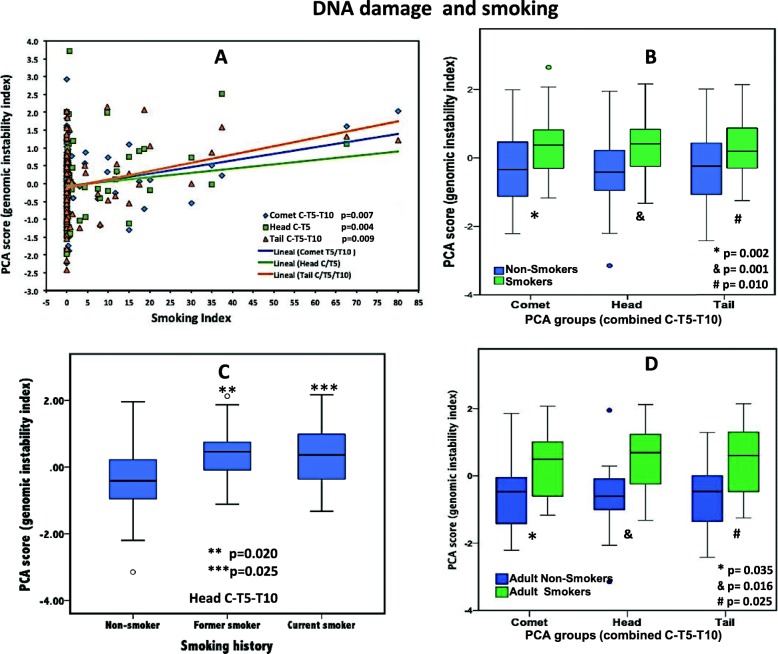
DNA damage and smoking. **a** Three PCA groups involving comet (blue diamonds), head (green squares), and tail (orange triangles) showed direct significant correlation with smoking index (*r* = .299, *p* = 0.007; *r* = .318, *p* = 0.004; and *r* = .287, *p* = 0.009, respectively). **b** Group comparison showing higher rates of DNA damage in smokers group (green bars); the three combined PCA that displayed significant differences (*p* ≤ 0.01) were the same for smoking index: comet, head, and tail (*p* = 0.002, *p* = 0.001, *p* = 0.010). **c** Former and current smokers had higher DNA damage rates represented by one group of PCA (head), *p* < 0.03 when compared to never smoker group. **d** Adult smokers (green bars) displayed higher DNA damage in three combined PCA groups (comet, head, tail) compared to their counterparts, non-smokers (blue bars), *p* = 0.035, *p* = 0.016, *p* = 0.025, respectively

The relationship between smoking (determined by the smoking index) and genomic instability was assessed by Spearman correlation. There was a statistically significant, positive correlation between the smoking index and the following PCA score groups: comet C-T5-T10 (*r* = .299, *p* = 0.007), head C-T5 (*r* = .318, *p* = 0.004), and tail C-T5-T10 (*r* = .287, *p* = 0.009) (Fig. 2a). Outliers were detected with Cook’s distance analyses, using the 4/*n* approach [17]. After taking them out, the correlation examination still resulted significantly; therefore, the whole real data were kept and are presented in the corresponding graph.

Independent samples *t* test confirmed significant differences in genomic instability between the groups of smokers and non-smokers in the comet C-T5-T10, head C-T5, and tail C-T5-T10 PCA groups (*p* = 0.002, *p* = 0.001, *p* = 0.010, respectively) with higher scores for smokers (Fig. 2b).

A PCA group (head C-T5-T10) mean score was significantly different for the three smoking category groups defined as former smokers, current smokers, and non-smokers. Greater DNA damage was found in former and current smokers compared to non-smokers (*p* = 0.020, *p* = 0.025, respectively) (Fig. 2c). Regarding age and smoking group, we ran an analysis for 6 groups (three age groups, subdivided into smokers and non-smokers each). The greatest difference lied on the adult category for the same three PCA mean score groups observed in smoker vs non-smoker comparison (*p* = 0.035, *p* = 0.016, *p* = 0.025) (Fig. 2d). We did not find significant differences in PCA scores between the groups of non-smoker young adults or non-smoker older adults versus their counterparts for the same age group. Notwithstanding, in the case of younger adults when individual parameters (non-PCA scores) were scrutinized, we found significant differences for two single components: comet intensity (*p* = 0.049) and head intensity (*p* = 0.023).

### Genotoxicity and lifestyle

#### Diet

Correlation analysis revealed that there was a significant positive association with the consumption of alcohol, energy drinks, and milk (higher frequency, higher genomic damage). On the other side, the foods that showed negative correlations were coffee, tea, sweeteners, tortillas, and nuts (Table 2).

**Table 2:** Correlation coefficient: food versus PCA score groups

Food	Correlation coefficient^a^	*p*	PCA group
Negative correlations			
Coffee	− .269	0.015	Comet C
	− .238	0.032	Comet C-T10
	− .241	0.031	Tail C
Tea	− .244	0.028	Comet T5-T10
	− .226	0.043	Head T5
	− .236	0.034	Head T10
Sweeteners	− .259	0.020	Comet T5
Tortillas	− .235	0.036	Comet C
	− .234	0.036	Head C
	− .226	0.044	Head C-T5
Nuts	− .248	0.026	Tail T10
Positive correlations			
Energizing drinks	.244	0.028	Comet C
Alcohol	.263	0.018	Head C-T5
Milk	.227	0.041	Comet T10

^a^Correlation was analyzed using two tails, Spearman, and weekly frequency consumption versus PCA scores

**Fig. 3:**
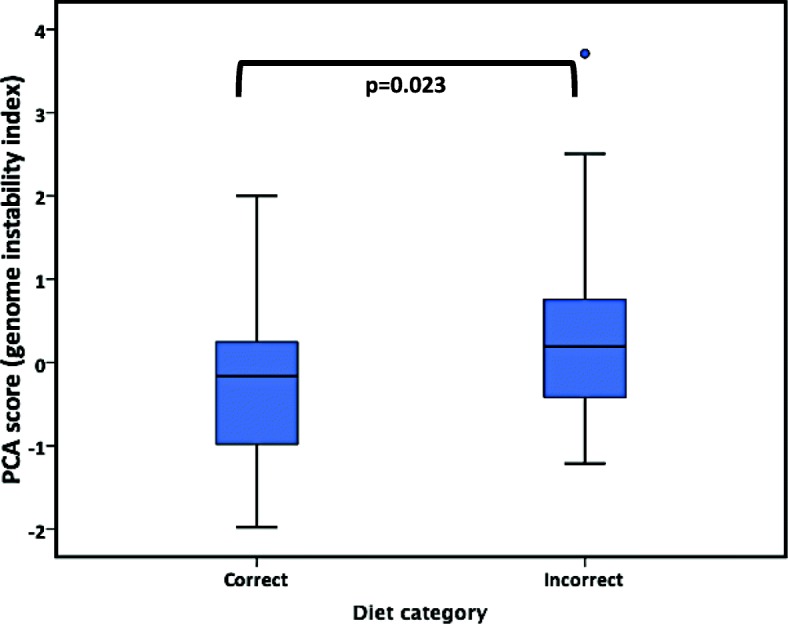
DNA damage and diet pattern. Subjects who were classified as having a “correct” diet, had decreased genomic damage as assessed by a head PCA group, *p* = 0.023

An independent sample *t* test was used to compare the means of PCA scores between correct and incorrect diets. The latter classification was integrated as recommended by Official Mexican Standard (NOM-043) [18]. The PCA group that exhibited significant differences corresponded to head C-T5, with higher rates of genomic damage displayed in the incorrect diet category (*p* = 0.023) (Fig. 3).

#### Exercise

**Fig. 4:**
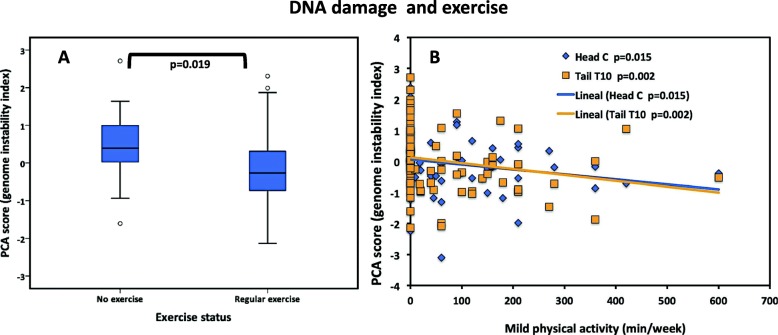
DNA damage and exercise. **a** Significant differences were observed between subjects who exercise versus those who do not exercise, with higher genomic damage (PCA tail group) for the latter, *p* = 0.019. **b** Mild physical activity time correlated significantly, inversely, with two PCA groups, head (blue diamonds) (*r* = − .270, *p* = 0.015) and tail (orange squares) (*r* = − .346, *p* = 0.002)

For this analysis, we classified the participants according to the level of physical activity they declared as stated by the International Physical Activity Questionnaire (IPAQ) [13]. The comparison of means showed higher scores in tail T10 for those participants that do not exercise in contrast to those that exercise regularly (*p* = 0.019) (Fig. 4a). As for the individual correlations, mild physical activity (i.e., walking with moderate pace) demonstrated a significant negative correlation with genomic instability rates: PCA head C group (*r* = − .270, *p* = 0.015) and PCA tail T10 (*r* = − .346, *p* = 0.002) (Fig. 4b). A small number of subjects who were classified under the intense physical activity category showed a positive significant correlation with PCA comet C-T5-T10 (*r* = .224, *p* = 0.045) and PCA tail C-T5-T10 (*r* = .245, *p* = 0.027).

#### Inactivity

**Fig. 5:**
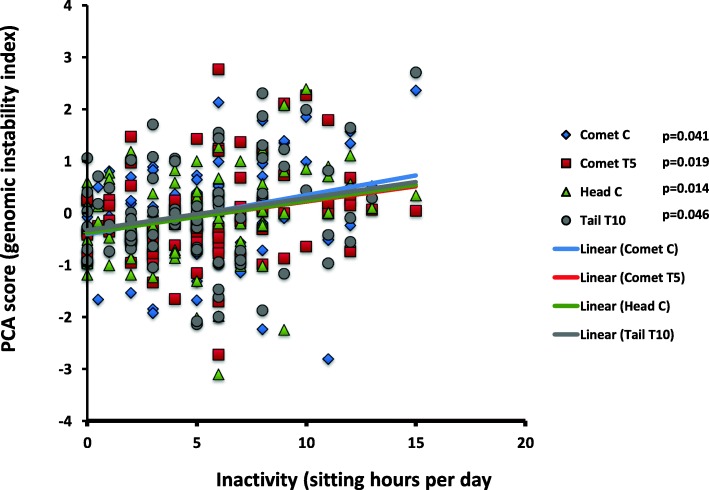
DNA damage and inactivity (sitting hours). Genomic damage was correlated significantly directly with 4 PCA group scores: comet C (blue diamonds) (*r* = .227, *p* = 0.041), comet T5 (red squares) (*r* = .260, *p* = 0.019), head C (green triangles) (*r* = 0.271, *p* = 0.014), and tail T10 (gray circles) (*r* = .221, *p* = 0.046)

Inactivity was surveyed as the average time spent sitting in hours throughout the day, and, interestingly, it displayed significant positive correlations with 3 PCA group scores: comet C (*r* = .227, *p* = 0.041), comet T5 (*r* = .260, *p* = 0.019), head C (*r* = 0.271, *p* = 0.014), and tail T10 (*r* = .221, *p* = 0.046) (Fig. 5).

#### Sleeping hours

We found a negative correlation between hours sleeping (at night) and two PCA groups: comet T5 (*r* = − .279, *p* = 0.012) and head T5 (*r* = − .320, *p* = 0.004), indicating an inverse relationship between sleeping time and genomic damage; that is to say the more time spent sleeping, the less genomic damage there is.

### Genotoxicity and family history of the disease

A comparison was completed to determine whether there were differences in genomic instability between the participants with a family history of cancer and those without any antecedent. Although no significant differences were found for the established PCA groups, there were some single parameters that displayed more damage when at least one grandparent had been diagnosed with cancer (comet tail DNA, *p* = 0.040; comet tail moment, *p* = 0.035; comet olive moment, *p* = 0.033).

### Genotoxicity and body composition

No significant results were observed when analyzing body mass index, muscle mass, fat percentage, hip, and waist girths. However, the arm girth showed moderate positive correlations with two PCA groups: head C-T5-T10 and head C-T5 (*r* = .262, *p* = 0.043, and *r* = .267, *p* = 0.040, respectively).

Regarding body composition, an interesting finding was the fact that non-smokers had a higher fat percentage than current smokers (*p* = 0.026) and former smokers (*p* = 0.015), using either the bioimpedance analysis or the relative fat mass (RFM) equation [19].

### Genotoxicity and radiation

A comparison was performed to determine whether recent exposure to radiation, in terms of X-ray affected genomic instability. We found higher rates of damage in participants who were exposed in a group of PCA, comet C (*p* = 0.009).

### Binomial logistic regression models

A binomial logistic regression analysis was performed to investigate whether or not the smoking index remained a significant predictor of the genomic damage when other factors were added to the model. To integrate “high” damage or “low” damage categories, we used a K-means cluster procedure in which all the comet parameters were analyzed and clustered into two categories. Three models were analyzed, in which the independent variables included age, diet, and exercise (Table 3). Two models showed that the smoking index was still a significant predictor (OR = 1.068, 95% CI 1.001–1.039; OR = 1.095, 95% CI 1.013–1.183) when inactivity was taken into account. The model 2 also showed that mild physical activity might indeed be significant for the less fragmentation outcome (OR = 0.993, 95% CI .987–.999). However, in the third model, where the categorical variable “exercise or not-exercise” was present, the smoking index was not found to be a significant predictor of damage indicating that smoking can be modulated by modifiable lifestyle factors like exercise. The models were tested for interactions between the significant terms, but no statistical significance was reached, indicating that predicted probabilities for genomic damage were dependent on the individual covariates included in the models.

**Table 3:** Binary logistic regression models

Model	Variables	Odds ratio	95% CI	*p* value
1. Omnibus test of model coefficients: 0.040 Hosmer-Lemeshow test: .910	Smoking index	1.068	1.001–1.139	*0.046*
	Age group (young adults)^a^			
	Age group (adults)	0.403	0.115–1.419	0.157
	Age group (older adults)	1.095	0.306–3.912	0.889
	Diet pattern (correct vs incorrect)	1.351	0.470–3.889	0.576
	Intercept	.694		0.409
2. Omnibus test of model coefficients: 0.008 Hosmer-Lemeshow test: .345	Smoking index	1.095	1.013–1.183	*0.023*
	Age group (young adults)^a^			
	Age group (adults)	0.312	0.076–1.281	0.106
	Age group (older adults)	0.928	0.231–3.733	0.916
	Diet pattern (correct vs incorrect)	1.862	0.588–5.896	0.290
	Inactivity	0.939	0.812–1.085	0.393
	Mild physical activity	0.993	0.987–.999	*0.030*
	Intercept	1.295		.698
3. Omnibus test of model coefficients: 0.034 Hosmer-Lemeshow test: .951	Smoking index	1.065	0.999–1.136	0.054
	Age group (young adults)^a^			
	Age group (adults)	0.358	0.098–1.309	0.120
	Age group (older adults)	0.961	0.260–3.547	0.952
	Diet pattern (correct vs incorrect)	1.348	0.459–3.960	0.588
	Physical activity or not	0.460	0.157–1.345	0.156
	Intercept	1.334		0.653

^a^Reference category for age group. Significant *p* values are shown in italics

The comparison of the empty model (M0) against model 1 (M1), model 2 (M2), and model 3 (M3) revealed significant differences and notable improvements of the model. Also, there were significant differences between models (Table 4), except for M1 versus M3.

**Table 4:** Comparison of binary logistic regression models

Comparison	Likelihood-ratio test	*p* value
M1 vs M0	9.82	*0.043*
M2 vs M0	17.67	*0.007*
M3 vs M0	11.41	*0.044*
M1 vs M2	7.85	*0.020*
M1 vs M3	1.59	0.207
M2 vs M3	6.26	*0.012*

Significant *p* values are shown in italics

## Discussion

With the assumption that genomic instability is one of the factors that trigger various types of cancer and other chronic diseases, we have designed a comprehensive analysis of the levels of genomic stability in a sample of smokers and non-smokers, with their corresponding assessment of anthropometry, lifestyle, and family history. Although it has been widely documented and accepted that smoking causes genomic damage, there have also been conflicting results when the assays include a phenotypic analysis, in which the extent of the damage is measurable visually. SCGE is a technique which is low cost and sensitive; however, the published results related to smoking have, sometimes, not confirmed the damage in a convincing manner. Some authors [20, 21] state that the lack of agreement upon the methodology is the main cause of variations in the results; in addition, most use only around 3 to 5 parameters when scoring genome instability, this is in contrast to our use of 16 variables added to the PCA procedure.

In the present study, we used those 16 parameters, and three treatments to add up to 48 measurements per participant, as retrieved from the image analysis open source, namely OpenComet [22]. In order to not discard any of the parameters arbitrarily, we undertook a PCA procedure for dimension reduction to integrate groups that load in one or combined categories of genomic instability. We refer to the latter as the fact that the score (coefficient or parameter) reported by the output of OpenComet is proportional to the damage. In other words, as the score increases (either for the whole comet body, comet head, or comet tail), the damage also increases (the greater the DNA fragmentation, the greater the scores for sizes and intensities) [23]. We assumed that the controversies reported were due to the fact that some variables may not have been taken into account. That prompted us to, systematically, include other aspects that can modify the outcome for genomic instability and analyze the variables in an individual manner (bivariate correlations and group comparisons) for age, smoking index, lifestyle (diet, exercise, inactivity), dietary pattern, and body composition with PCA groups. Also, according to our hypothesis, we confirmed that the association between genomic instability and smoking could be modulated and smoking index loses its predictive value when those co-variables were included in a model of logistic regression.

We will discuss every analyzed variable and the results obtained with genomic damage scores, as well as the results of logistic regression models.

In the first place, our results consistently showed a significant positive association between smoking index and some PCA groups. In this regard, some studies have failed to find a significant difference between smokers and non-smokers using SCGE or significant association dependent on the score methods [12, 24]. On the other hand, some others have concluded that smoking can cause DNA instability when analyzed on peripheral blood cells as measured by the comet assay [21, 25]. We actually found a significant correlation with the smoking index, which has indeed been reported to have an involvement in the development of some diseases such as chronic obstructive pulmonary disease and lung cancer. The reports have established that high smoking indexes were associated with a greater risk of contracting those pathologies [26]. Nonetheless, it has been shown that DNA damage can be reversible when people quit the habit [27], and cessation can reduce all-cause mortality up to 30% [28]. We have detected higher genomic damage in former smokers than never smokers, and it has been reported the identification of epigenetic modification in those who quit for up to 22 years [29]. We demonstrated, as expected, that DNA damage was associated with age in agreement with other studies [30] which have shown higher scores of comet assay parameters as age increases. However, to our knowledge, this is the first study to analyze the comet assay using PCA scores and the smoking habits by age group.

An interesting outcome was the fact that the highest differences of DNA damage were present in smoking adult group vs non-smoking adult group, but no differences were found in their younger or older counterparts. In this regard, there is one study that did not show any significant differences between smokers and non-smokers within the same age group of our younger adults [31]. Another study also did not report any significant differences but the authors did not specify the mean age of smokers and non-smokers, and certainly, no older adults participated in the study [32]. These discrepancies, lead us to consider other factors that could be influencing the outcomes of phenotypic analysis for genomic damage, apart from the fact that it is known that younger people have higher rates of DNA repair while older people exhibit higher basal DNA damage [30]. Several of those factors have been analyzed in some of the studies previously mentioned and so have we in the present report. In the case of physical activity, we found that those subjects who did not perform any kind of workout exhibited higher rates of genomic instability; moreover, a positive significant relationship was observed with the number of hours spent sitting (inactivity). Those findings agreed with other studies showing that mild or moderate physical activity reduces the risk of diseases [33] and sitting time increases the risk of death, independently of physical activity [34]. We also found that high-intensity workout is associated with greater instability, as it has been demonstrated by others [35, 36]. Among other analyzed variables related to lifestyle, we found less damage in people who reported more sleeping hours (negative correlation). This association has been reported in two studies with rat models [37, 38] specifically designed to quantify the damage with SCGE and in a very recent study in humans that used a different gene expression approach [39]. In the case of genomic instability and some groups of food, it is well known that dietary factors exert changes in DNA, either protecting it or damaging it. In our case, coffee, tea, sweeteners, tortillas, and nuts presented a negative association with genomic damage; in other words, less damage was found. A note about sweeteners is the fact that this diet component showed a significant positive correlation with coffee consumption (*r* = .250, *p* = 0.024), which may partly explain the inverse correlation with genomic damage. There exists widely accepted evidence that coffee and tea are a rich source of antioxidants and could contribute to lower the DNA damage [40–42]. As for nuts, there is a publication [43] that demonstrated some preventive effects on DNA damage caused by smoking an assessed by SCGE. Meanwhile, for tortillas, we did not find any report other than the fact that niacin enhances its bioavailability because of the process that goes into the production of tortillas (alkali treatment), so people who consume this food generally do not have niacin deficiency [44].

Even though it has been postulated that being overweight (and obesity) is associated with an increased risk for cancer, we only found a significant association with arm girth and DNA damage. In this regard, there are some contradictory findings from no significant association of DNA repair capacity and weight loss [45] to an increased level of DNA damage in tumor cells and PBMC obtained from endometrial cancer associated with BMI [46].

Precisely, in the latter paper, the authors also found that DNA damage increases in volunteers with a family history of cancer, the same as in our study. However, in our case, we only found significant differences for individual parameters (not in PCA groups) in participants who said to have at least one grandparent who was diagnosed with any type of cancer.

Among other significant findings, we detected that even small occasional exposure to radiation (X-ray) was positively correlated with one group of PCA, the fact that has been previously reported in radiology personnel [47].

Finally, when we integrated the logistic models, the smoking index was still associated to an increased risk of pertaining to the high fragmentation group by around 7 to 9%, adjusted for age, diet, and inactivity; however, when a category of exercise or not exercise was present, the smoking index lost its significance. The models were significantly different when compared to the baseline, and the addition of the variables related to physical activity showed significant improvements to the fit. However, no differences were found when mild physical activity and inactivity were substituted for the categorical presence or absence of exercise.

We believe that our study has strengths since we used all the comet assay parameters, without choosing only the ones which were significant but simplifying the complexity in high-dimensional data while retaining trends and patterns, as the PCA procedure implies.

We acknowledge that the correlation coefficient reached a small, yet a significant effect size; however, the bivariate analysis was performed with the scores of an integrated multivariate analysis (PCA) in which all of the parameters retrieved from SGCE image analysis were included, and in most of the cases, more than one PCA group displayed consistent results.

Among other factors, genomic instability might arise from and intrinsic impairment of DNA repair systems [48], and as it has been discussed previously, some lifestyle factors or environmental exacerbate the burden of chronic degenerative diseases like diabetes or neurological diseases [49, 50]. For the present study, we reckon that one of the limitations was that we did not scrutinize the DNA repair systems which are constantly active and receive influence from various exogenous and endogenous signals that can change the rate of damage. In connection with the exogenous signal point, there is a review that evaluated 28 studies in terms of DNA damage related to occupational and environmental exposure to miscellaneous chemicals, and 75% of them showed some type of genomic damage, probably due to a defective homeostasis of metal ion which can interfere with DNA repair [51]. Prospectively, there should be more designs in this regard to possibly perform again the PCA system in bigger samples which is one of the most powerful tools in the data analysis.

In general, even though SCGE is a cost-effective approach to screen genomic damage, and it has been used widely in many contexts, it still holds some limitations, which span from developing technical skills and standardizing the method in the laboratories to the statistical analysis. In the first case, the preparations should be the cleanest possible and the image analysis should be operated by a trained technician that must be blinded to the nature of the specimen origin. A very carefully experimental plan should be planned ahead to avoid time-consuming failures. Another constraint regarding the use of the SCGE is the analysis of the damage at a microscopic level, leaving the analysis at a phenotypic evaluation. The statistical analysis can be an issue; however, most of the outputs from the image analysis software retrieve continuous data which are a valuable input in parametric inferential statistics.

## Conclusion

In conclusion, we evaluated genomic instability associated with smoking, and other lifestyle factors such as diet, exercise, and age, using PCA scores to evaluate the phenotypic analysis of damage and binomial logistic regression analysis. The punctuation of genomic damage could be incorporated into a risk predictor model that takes into account the covariates that can modify the predicted probability of smoking index for genomic damage. The phenotypic analysis of detectable damage by SCGE could be conditional of age group.

## Methods

### Study subjects

A sample size of 85 subjects aged 18–95 years was selected for the study. The calculation for the sample size was performed to obtain the minimum sample size, following the procedure for determining whether a correlation coefficient differs from zero. The assumptions included a statistical power of 80% and an expected correlation coefficient of 0.3 as a medium effect size [52]. Their participation was voluntary, and all of them provided written informed consent. However, 3 subjects retired their consent, leaving 82 subjects with completed tests. This sample size led to a still reasonable statistical power of 78%. Our inclusion criteria were a minimum age of 18 years old and have been residing in the Yucatan Peninsula for at least a year. In addition, we defined our sample to allow for two groups to form based on the smoking habits, so we would have half of the participants classified as smokers and the other half as non-smokers. The sampling strategy was as follows: after an open call to participate, with a deadline, our sample was selected from a list of registered volunteers and the final participants were chosen using a systematic probabilistic sampling.

We did not exclude participants based on whether they had any disease, were under any medication, or suffered any medical condition, as it was an open call and we aimed to integrate a more representative sample. At baseline, participants completed a comprehensive-specific questionnaire (collecting information on sociodemographic characteristics, lifestyle factors, and history of major diseases among others). The ethics committee of the School of Medicine at University Anahuac Mayab approved the study (MED/066/17).

### Questionnaire

The administered survey to each participant covered five domains, namely smoking habits, nutrition, physical activity, family history of cancer, and miscellaneous data.

The first domain explored the smoking status, in order to define a categorical variable based on current or past smoking habits. We defined non-smokers as those who have never smoked and smokers who have smoked in the past or are currently into smoking. We also determined the smoking index for each individual as a unit for measuring cigarette consumption over a long period in current and former smokers. It was calculated using the following formula: smoking index = cigarettes smoked per day multiplied by the smoking time in years divided by 20. In this regard, the published literature refers to this term as pack-years, [26]; however, there are some reports that mention this indicator as smoking index [53, 54] as well as is denoted in that way in the National Clinical Practice Guidelines (MEX) [55].

The nutritional aspect was explored through an adapted CDC’s National Health and Nutrition Examination Survey (NHANES), which was applied to participants to examine their diet over the last 6 months [56]. Based on the collected information, a certified nutritionist classified the dietary pattern of each participant as “correct” or “incorrect” according to the NOM-043 [18]*.* In this document, a correct diet is described as complete, balanced, innocuous, sufficient, varied, and adequate. So, to qualify as a correct diet, the participant must include the three food groups in every meal: fruits and vegetables, legumes and food of animal origin, and finally cereal, with adequate proportions.

Regarding physical activity, a modified IPAQ [13] was applied to define the characteristics of the physical activity for each participant. It classifies the type of exercise as intense, moderate, or mild according to the frequency of activity in terms of minutes of exercise per day and number of exercise days per week. We also recorded hours of sleep and inactivity (measured as hours spent sitting).

The fourth domain was designed to investigate whether the participants had a family history of cancer, since it is widely accepted that some genomic alterations have an inherited component. We only considered parents and grandparents for this variable that was categorically recorded as the presence or absence of history of cancer in any of the aforementioned relatives.

We also registered other aspects (“miscellaneous” domain), in which we looked for information on the personal and family history of the disease (different from cancer), exposure to radiation, passive smoking, exposure to smoke other than cigar, prescribed drug consumption, recreational drug use, and antioxidant supplementation.

### Body composition evaluation

Weight and height were measured to the nearest 100 g and 0.1 cm, respectively, during a physical exam by trained nutritionists according to strict standard operating procedures, using SECA 875 electronic scales (SECA, UK) and SECA 217 stadiometer (SECA, UK). Additionally, waist, hip, and arm girths were measured in centimeters with Gulick II Plus Tape Measure (Performance Health, Chicago, USA). The body mass index was calculated with Quetelet’s formula [57]. A bioelectrical impedance analysis was performed with a portable device (InBody 270, Seoul, Korea) to determine water content, muscle, and bone mass percentages. Additionally, we used the recently RFM equation to estimate whole-body fat percentage [19].

### Determination of genomic instability

SCGE was used to detect DNA damage in white blood cells and was performed as described previously [23]. Briefly, a peripheral blood sample was obtained from each participant, layered onto a volume of histopaque, and centrifuged according to the manufacturer’s recommendations. After centrifugation, a volume of the PBMC buffy coat was aspirated, washed, and resuspended with phosphate-buffered saline. Cell count was performed using an aliquot of diluted cells, using a hemocytometer. The cell suspension was adjusted to a final dilution of 3 × 10^5^ cells per milliliter and split for 3 treatments: one with PBS as a control (C), the second, and third ones were added 5% (T5) and 10% (T10) hydroxide peroxide (H_2_O_2_) from a stock solution of 3%, to induce cell damage (oxidation), so we had three different treatments for each subject. The cells were treated for 20 min at 37 °C. Then, each dilution was included in low melting point agarose mini-gels and layered onto normal melting point agarose-primed slides. Once the mini-gels dried out, they were immersed in a lysis buffer to expose nuclear material. Afterwards, alkaline electrophoresis was performed at 25 V (constant) for 40 min (Thermo Scientific™ Owl™ Horizontal Gel Electrophoresis System, MA, USA). Finally, a neutralizing buffer was added and the gels were stained with an intercalating agent (ethidium bromide). The slides were scrutinized with fluorescence microscopy (Zeiss Imager.A2, camera AxioCam Icc1, Germany) to identify nuclear (DNA) damage. The image analysis was carried out with specialized image acquisition software (ZEN 2 lite, blue edition) and to perform the genomic damage measurements (OpenComet v1.3.1) [22]. For each component of the image (head, comet body, and tail), 16 parameters were analyzed and retrieved by the software OpenComet, per treatment, being 48 parameters in total for each participant. In total, we analyzed 82 biological samples. The technician who analyzed the images was blinded on the characteristics of the individual whose biological samples were under study.

### Statistical analysis

The analysis was carried out (using IBM® SPSS® Statistics, v. 24 for Windows) with all of the 16 comet assay parameters per cell suspension treatment (C, T5, T10), to add up a total of 48 variables per participant. To establish the internal consistency and the correlation between them, we ran a Cronbach’s alpha, which displayed a value of 0.812. Moreover, we confirmed that the 48 variables exhibited a significant correlation (*p* < 0.05) between them, so we can state that a high value is coherent with more damage, as detected with induced oxidation level (T5 and T10).

Every variable was tested for data distribution, and normality was assessed with Shapiro-Wilk’s test [58]. However, because of the sample size, in the case of correlation, two-sided Spearman correlation was applied. For comparisons, if the testing variable showed a normal distribution, we used independent samples *t* test or ANOVA. If the distribution was non-normal, for two independent samples, we used Mann-Whitney *U* test, and for more than two groups, we used Kruskal-Wallis test.

### Principal component analysis

We performed a dimension reduction to integrate valid patterns per treatment; therefore, we applied PCA and rotation to derive genomic damage. A correlation matrix was constructed to assess the correlation between genomic damage. The Kaiser-Meyer-Olkin test (≥ 0.6) and Bartlett’s test of sphericity (*p* value < 0.05) were applied to verify whether the PCA assumptions were met [59]. Varimax rotation was applied to obtain orthogonal factors. Genomic damage groups that showed factor loadings greater than 0.3 were considered to have strong associations with that factor. The number of factors that best represents the data was based on the screen plot and eigenvalues above 1.5. Genomic damage patterns were named according to the segment of the comet retrieved by the software, OpenComet, and the treatment under scrutiny, that way the genomic damage parameters were defined as comet, head, or tail, with their 3 treatments each: control, 5% (T5), and 10% (T10) of H_2_O_2_ (from a stock solution of 3%) for each segment. The PCA groups are named individually (according to the part of the comet and their treatment or as a combined score composed of comet body, head, and tail in their different treatments). Table 5 shows the nomenclature for each PCA group.

**Table 5:** Nomenclature of PCA groups

Abbreviated name of PCA group	Extended name	Variables included
Comet C	Total comet body without treatment	Comet area, comet length, comet DNA (pixels and percent)
Comet T5	Total comet body with 5% H_2_O_2_	
Comet T10	Total comet body with 10% H_2_O_2_	
Head C	Comet head without treatment	Head area, head DNA, head intensity, head length
Head T5	Comet head with 5% H_2_O_2_	
Head T10	Comet head with 10% H_2_O_2_	
Tail C	Comet tail without treatment	Tail moment, tail length, tail area, tail DNA (pixels and percent)
Tail T5	Comet tail with 5% H_2_O_2_	
Tail T10	Comet tail with 10% H2O2	
Comet C-T5-T10	Combined scores of total comet body with and without treatment	Comet intensity
Head C-T5-T10	Combined scores of comet head with and without treatment	Head intensity
Tail C-T5-T10	Combined scores of comet tail with and without treatment	Tail intensity

### Binomial logistic regression

Firstly, we defined our dependent variable as “higher damage” (higher fragmentation of nuclear content) or “lower damage.” In order to do so, we used a K-means cluster strategy, in which all of the 48 observations (per subject) were input in order to find scores that cluster into two groups: higher and lower damage. Afterwards, we confirmed that the comet assay parameters exhibited a significant difference for the two groups (Table 4) with higher scores for that group labeled as higher damage.

Secondly, our aim was to build a model to ascertain the effects of smoking index, age, and lifestyle (diet, exercise, inactivity) on the likelihood that participants have “higher” genomic damage. From a K-means cluster analysis, we obtained two different clusters, which we considered as the dependent variable for the binomial logistic regression by using the method “enter,” with the following predictor variables taken into account for each model: for model 1, smoking index, age, and diet; for model 2, smoking index, age, diet, inactivity, and mild physical activity; and for model 3, smoking index, age, diet, and exercise or not. The three models were statistically significant as follows: model 1, *X*^2^ (4) = 10.044, *p* = 0.040; model 2, *X*^2^ (6) = 17.451, *p* = 0.008; model 3, *X*^2^ (5) = 12.084, *p* = 0.034.

The models explained 15.6%, 25.9%, and 18.9% of the variance in the class of higher damage, (based on Nagelkerke *R*^2^), for models 1, 2, and 3, respectively, and correctly classified 63%, 67.9%, and 61.7% (models 1, 2, and 3, respectively) of cases. No multicollinearity was detected in any of the models using variance inflation factor and tolerance. This is none of the variables included in the models showed a VIF less than 3, all tolerance values were higher than 0.2, the condition indexes were smaller than 15, and there were not two or more variables with an eigenvalue greater than 0.90. Interactions between covariates were carried out in those models which fulfilled a significant omnibus test (*p* < 0.05) and an appropriate Hosmer-Lemeshow Goodness-of-Fit test (*p* > 0.05). The number of included covariates (or interaction terms) was based on the rule of ≥ 10 events per variable [60] for all of the models tested. The final models were compared using the likelihood-ratio test.

## Data Availability

The datasets generated and/or analyzed during the current study available from the corresponding author on reasonable request.

## Abbreviations

BMI: Body mass index
IPAQ: International Physical Activity Questionnaire
NHANES: National Health and Nutrition Examination Survey
NOM-043: Official Mexican Standard
PBMCs: Peripheral blood mononuclear cells
PCA: Principal component analysis
RFM: Relative fat mass
SCGE: Single-cell gel electrophoresis
